# The Anti-Cancer Activity of the Naturally Occurring Dipeptide Carnosine: Potential for Breast Cancer

**DOI:** 10.3390/cells12222592

**Published:** 2023-11-08

**Authors:** Salvatore Maugeri, Jay Sibbitts, Anna Privitera, Vincenzo Cardaci, Lucia Di Pietro, Loredana Leggio, Nunzio Iraci, Susan M. Lunte, Giuseppe Caruso

**Affiliations:** 1 Department of Drug and Health Sciences, University of Catania, 95125 Catania, Italy; 2 Ralph N. Adams Institute for Bioanalytical Chemistry, University of Kansas, Lawrence, KS 66047, USA; 3 Department of Chemistry, University of Kansas, Lawrence, KS 66047, USA; 4 Department of Biomedical and Biotechnological Sciences, University of Catania, 95123 Catania, Italy; 5 Scuola Superiore di Catania, University of Catania, 95123 Catania, Italy; 6 Vita-Salute San Raffaele University, 20132 Milano, Italy; 7 Department of Pharmaceutical Chemistry, University of Kansas, Lawrence, KS 66047, USA; 8 Unit of Neuropharmacology and Translational Neurosciences, Oasi Research Institute-IRCCS, 94018 Troina, Italy

**Keywords:** carnosine, cell proliferation, cell cycle, cell metabolism, breast cancer, metastases

## Abstract

Carnosine is an endogenous dipeptide composed of β-alanine and L-histidine, possessing a multimodal pharmacodynamic profile that includes anti-inflammatory and anti-oxidant activities. Carnosine has also shown its ability to modulate cell proliferation, cell cycle arrest, apoptosis, and even glycolytic energy metabolism, all processes playing a key role in the context of cancer. Cancer is one of the most dreaded diseases of the 20th and 21st centuries. Among the different types of cancer, breast cancer represents the most common non-skin cancer among women, accounting for an estimated 15% of all cancer-related deaths in women. The main aim of the present review was to provide an overview of studies on the anti-cancer activity of carnosine, and in particular its activity against breast cancer. We also highlighted the possible advantages and limitations involved in the use of this dipeptide. The first part of the review entailed a brief description of carnosine’s biological activities and the pathophysiology of cancer, with a focus on breast cancer. The second part of the review described the anti-tumoral activity of carnosine, for which numerous studies have been carried out, especially at the preclinical level, showing promising results. However, only a few studies have investigated the therapeutic potential of this dipeptide for breast cancer prevention or treatment. In this context, carnosine has shown to be able to decrease the size of cancer cells and their viability. It also reduces the levels of vascular endothelial growth factor (VEGF), cyclin D1, NAD^+^, and ATP, as well as cytochrome c oxidase activity in vitro. When tested in mice with induced breast cancer, carnosine proved to be non-toxic to healthy cells and exhibited chemopreventive activity by reducing tumor growth. Some evidence has also been reported at the clinical level. A randomized phase III prospective placebo-controlled trial showed the ability of Zn–carnosine to prevent dysphagia in breast cancer patients undergoing adjuvant radiotherapy. Despite this evidence, more preclinical and clinical studies are needed to better understand carnosine’s anti-tumoral activity, especially in the context of breast cancer.

## 1. Introduction

The terms cancer and tumor indicate a pathological condition characterized by uncontrolled proliferation of abnormal cells that have the ability to infiltrate organs and tissues. Furthermore, cancer is characterized by an aberrant interaction with the immune system and represents one of the most dreaded diseases of the 20th century. In particular, breast cancer is a potentially deadly disease, especially if it is not detected and treated in time. It is now the most common non-skin cancer among women. The five-year survival rate (i.e., the percentage of people alive five years after they were diagnosed with or started treatment for a disease) for patients diagnosed with breast cancer at stages I and II, while it is still localized, is 98%. However, the rate significantly decreases for those diagnosed at stage III (72%) and stage IV (22%) [1]. Currently, it is predicted that there are 268,000 new diagnoses per year in the USA, representing approximately 30% of all new cancer cases in women. Breast cancer is the second most common cause of cancer-associated deaths in women, and in the US annual breast cancer deaths are in excess of 41,000 (or approximately 15% of all cancer-associated deaths for women) [2].

Breast cancers are classified based on the molecular subtype. The most common include the following: (i) luminal A and luminal B, characterized by low (luminal A) or high (luminal B) levels of the protein Ki-67, while both expressing estrogen receptor (ER) and progesterone receptor (PR), but not the human epidermal growth factor receptor (Her2); (ii) Her2 positive, expressing Her2, but not ER or PR; and (iii) triple-negative breast cancer (TNBC), expressing none of the above-mentioned receptors. These subtypes are all significantly different in terms of metastases, prognosis, and treatment methods [3]. In this review, we analyzed the literature with particular regard to breast cancer treatment using carnosine and its potential therapeutic effects. Carnosine is a naturally endogenous dipeptide that is present at high concentrations in several tissues, particularly in the cardiac and skeletal muscles [4,5], as well as in the brain [6]. The concentrations of carnosine in cells are regulated via the activity of two different carnosinases [7]. This dipeptide possesses different protective abilities and can exert a multimodal mechanism of action [8], including antioxidant, anti-inflammatory, anti-aggregant, and anti-tumoral activities. Although it has been suggested that carnosine is involved in cell proliferation, cell cycle arrest, apoptosis, and even in the glycolytic energy metabolism of certain tumor cells, the molecular mechanisms of the anti-neoplastic activities of this dipeptide are still not completely understood and need to be further investigated.

## 2. Carnosine: History and Biological Activities

### 2.1. Carnosine’s Metabolism

**Figure 1:**
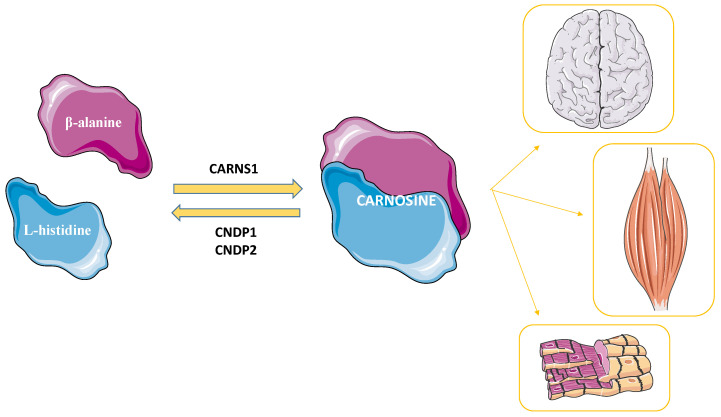
Carnosine’s synthesis and metabolism occur through the activities of carnosine synthase 1 (CARNS1) and the carnosinases (carnosine dipeptidase 1 (CNDP1) and carnosine dipeptidase 2 (CNDP2), respectively). The tissues containing the highest levels of carnosine are represented. Created with https://smart.servier.com (accessed on 10 April 2023).

Carnosine was discovered by Gulewitsch and Amiradžibi more than 100 years ago (1900), in the Laboratorium der Universität Charkow, as an abundant non-protein nitrogen-containing compound of meat; for this reason, it was given the name “carnosine”, from the Latin *caro*, *carnis* (meat) [9]. It is a naturally occurring endogenous dipeptide synthesized by the enzyme carnosine synthase 1 (CARNS1) from the amino acids β-alanine (synthesized in the liver) and L-histidine (external source) [10,11] (Figure 1).

The levels of this dipeptide in the body are regulated through the activity of two carnosinases that catalyze the hydrolysis of carnosine into its constituent amino acids, namely the serum-circulating carnosine dipeptidase 1 (CNDP1) [12] and the cytosolic carnosine dipeptidase 2 (CNDP2) [13].

### 2.2. The Multimodal Mechanism of Action of Carnosine

Numerous studies have reported the ability of the dipeptide carnosine to exert different biological and physiological roles through its well-known multimodal mechanism of action. Most of the carnosine (about 99%) is localized in muscle tissues [4], which is why there are a plethora of research studies that have investigated its activities in muscles, and in particular the benefits to the athletic performance related to carnosine (or its precursor β-alanine) supplementation. In this context, carnosine has been shown to facilitate the detoxification of muscles from lactic acid accumulation [14], to act as an endogenous antidote [15], and to improve both cellular ion/proton exchange [16], as well as contraction and mechanical work production in muscles [17,18]. Muscle relaxation rates and endurance exercise are additional activities that can be positively modulated via carnosine [19,20,21,22,23,24,25].

Even though the highest concentrations of carnosine can be found in muscles and brain, this dipeptide also exhibits biological activities in other areas of the body. In fact, it functions as a neurotransmitter [26] and enhances cell energy metabolism mediators (e.g., high-energy triphosphates and nicotinic coenzymes) in macrophages and microglia by rescuing and/or increasing basal levels [27,28,29,30] and the immune system [31]. Also, carnosine functions as a modulator of the metabolism of nitric oxide (NO) and its related species in immune cells [32,33,34], as an enhancer of the activity and migration of stem cells [31], a promoter of wound healing [35], and a regulator of osmotic pressure [36]. Carnosine also plays a role as an anti-glycation and anti-aging agent [37,38], as well as a chelator of heavy metals [39,40]. Moreover, carnosine has been shown to modulate the glutamatergic system through the up-regulation of glutamate transporter 1, causing a reduction in glutamate levels in the central nervous system [41]. Oxidative stress, defined as an aberrant over-production of reactive species that overcomes the antioxidant machinery, and inflammation have been increasingly recognized as playing pivotal roles in the pathogenesis of numerous diseases [42,43]. These previously described properties, along with the well-known antioxidant, anti-inflammatory, and anti-aggregant activities, suggest a therapeutic potential of carnosine in diseases characterized by oxidative stress, inflammation, and/or aberrant protein aggregation, such as Alzheimer’s disease, type 2 diabetes mellitus, and cardiovascular diseases [7]. Starting from its ability to selectively inhibit transformed cell proliferation [44], carnosine has been shown to be able to inhibit the proliferation of different types of cancer, such as gastric, colon, and ovarian cancer [45,46,47], along with breast cancer, which is discussed in more detail later in this article.

## 3. Pathophysiology of Breast Cancer

Cancer incidence has been rising alarmingly due to our changing lifestyle, habits, increased exposure to carcinogens, and increased life expectancy. It has been estimated that one in four people will be diagnosed with cancer in their lifetime. The risk of cancer is even higher in those individuals whose immune system is suppressed due to a variety of causes, including chronic stress, old age, chronic debilitating diseases, previous use of chemotherapy, and abuse of drugs, such as analgesics, antibiotics, and corticosteroids, or alcohol [1].

The malignant transformation of a cell is characterized by the accumulation of mutations in specific classes of genes, such as proto-oncogenes and onco-suppressor genes, which together only constitute a small proportion of the full genetic set. However, mutations in these genes, in particular, account for most of the uncontrolled cell proliferation seen in cancer. Furthermore, alterations in other classes of genes may also participate in the onset of cancer specifically by enabling aberrant proliferation that can ultimately lead to metastasis to other tissues throughout the body [48,49].

In recent years, researchers have expanded their interests to stem cells to develop novel therapies for cancer. Stem cells renew themselves and are able to differentiate into different types of mature cells. However, certain genetic mutations within stem cells can alter the differentiation process, leading to stem cell transformation. Additionally, the innate plasticity of stem cells may result in transformed clones becoming more aggressive and adaptive, and thus resistant to cancer treatments. However, it is not clear yet if stem cells (or stem-like cells) are capable of causing cancer progression [50]. Another recent theory involves oncohistones, which are histones with high-frequency point mutations that are associated with tumorigenesis [51,52].

**Figure 2:**
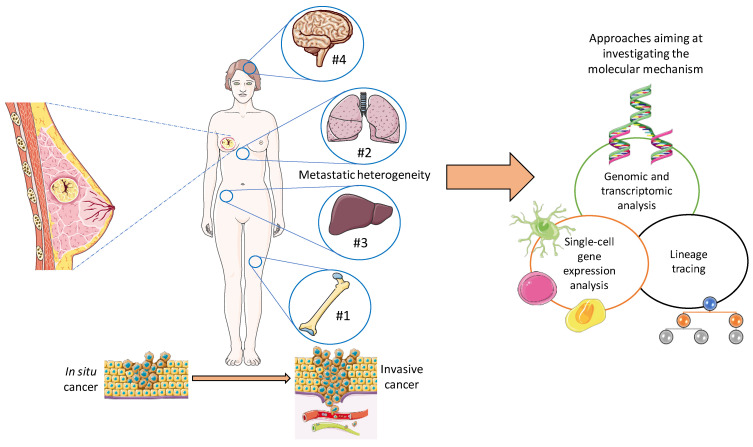
The percentage of patients affected by breast cancer who could develop metastases after diagnosis and primary tumor treatment is 20–30%. Breast cancer has demonstrated a metastatic propensity towards various organs, such as the bones (1st most targeted site, 75% of metastatic cases), lungs, liver, and brain. New approaches are available to investigate the molecular mechanism leading to metastatic heterogeneity, including genomic and transcriptomic analysis, single-cell sequencing strategies, and lineage tracing. Created with https://smart.servier.com (accessed on 10 April 2023).

In this review, particular attention was given to breast cancer, the most common nonskin cancer among women, accounting for an estimated 15% of all cancer-related deaths in women [2]. Currently, through advances in early diagnosis and treatment strategies, the prognosis of breast cancer patients has improved. As mentioned above, breast cancer can generally be classified as being one of four types based on molecular subtypes: luminal A, luminal B, Her2 positive, and TNBC [3]. Particularly, therapies targeting Her2 have been used, with relative success for Her2-positive breast cancer [53,54,55]. Her2, a tyrosine receptor kinase associated with cancer cell aggressiveness, has been reported to be over-expressed in 20–25% of breast cancer cases [56,57,58]. Conversely, TNBC represents the most aggressive breast cancer and accounts for 15–20% of breast cancers, and unfortunately current diagnostics are usually only able to detect it at more advanced stages. This delay in diagnosis results in a significantly higher rate of recurrence and a concomitant lower survival rate [59,60]. Additionally, TNBC does not have an effective targeted therapy as of yet. The recurrence rate of metastasis is also increased, and the patients’ prognosis is closely related to it [61]. For most types of cancer, the onset of metastasis marks the beginning of the final stage, and unfortunately metastasis is a sophisticated process involving a plethora of cellular mechanisms, including immune evasion as well as regulation of the tissue microenvironment. Most forms of cancer also require cells to undergo an epithelial–mesenchymal transition to become metastatic [62,63]. In terms of percentage, the portion of breast cancer patients that may develop metastases after diagnosis and primary tumor treatment is 20–30%, and 90% of cancer-related deaths are caused by metastasis [64] (as displayed in Figure 2).

More than 80% of patients affected by breast cancer who do not present metastasis have a survival rate of 5 years overall [65]. Nevertheless, distant metastasis can lead to a tragic reduction of the survival rate to 25% [66]. Breast cancer has demonstrated a metastatic propensity towards various organs, such as the bones, lungs, liver, and brain. This is known as metastatic heterogeneity, and can result in different responses to treatment and patient prognosis. Bone metastasis represents 75% of cases [67], with a five-year survival rate of 22.8% [68]. Lungs are the 2nd most targeted site [69], with a five-year survival rate around 16.8%, followed by liver metastasis, which, unfortunately, presents a lower survival rate relative to loco-regional bone and lung relapse, with an expected five-year survival rate of 8.5% [70]. Around 15–30% of patients affected by metastatic breast cancer develop brain metastases, which leads to a major limitation regarding the quality of life and life expectancy with a further reduced survival rate [71,72,73]. The molecular heterogeneity of metastatic breast cancer is possibly one of the main reasons why a significant number of therapies fail. Therefore, more research into the mechanisms that give rise to this molecular heterogeneity would ensure more effective metastasis-targeting agents and improve the life expectancy of patients. With recent technological advancements, many approaches have become available to expand our knowledge about the underlying molecular mechanism(s), including genomic and transcriptomic analyses [74,75,76,77], single-cell sequencing strategies [78], and lineage tracing [79]. Another expanding research approach has been focused on improving models of metastasis in animals and on harvesting new tumor cell lines with different organotropisms [80,81,82,83]. Finally, functional experiments will help to identify novel molecules related to this metastatic heterogeneity [84].

## 4. Anti-Tumoral Activity of Carnosine: Possible Mechanisms

**Figure 3:**
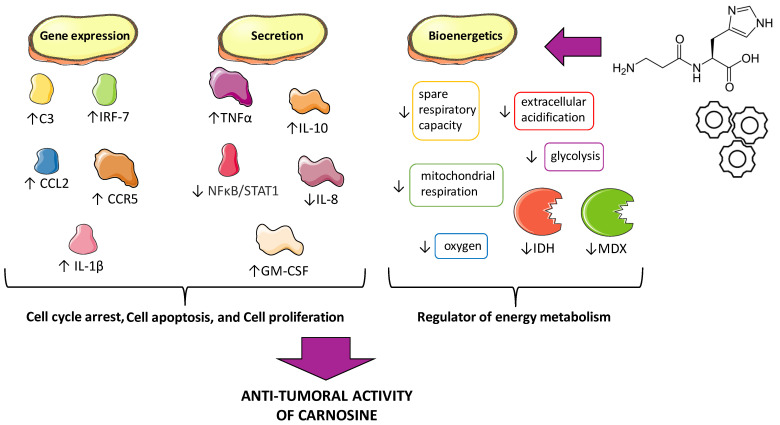
Carnosine exerts its anti-tumoral activity by modulating the expression of genes and/or the secretion of factors related to tumor growth, including cell proliferation, apoptosis, and the cell cycle, as well as modulating the activities related to bioenergetics/cell energy metabolism. In particular, this dipeptide is able to modulate the expression and/or release of chemokines and cytokines (e.g., CCL2 and IL-1β), trophic factors (e.g., GM-CSF), and pro-inflammatory pathways (e.g., NF-κB), as well as the activities of different enzymes (e.g., IDH and MDX). Created with https://smart.servier.com (accessed on 10 April 2023).

It is now known that carnosine is involved in the processes of cell proliferation, cell cycle arrest, apoptosis, and even in the glycolytic energy metabolism of certain tumor cells (Figure 3); however, the molecular mechanisms of the anti-neoplastic activities of this dipeptide remain unclear.

### 4.1. Cell Proliferation, Cell Cycle Arrest, and Apoptosis

Prakash et al. [85] investigated the effects of carnosine on several cancer cell lines, both in term of proliferation as well as gene expression and cytokine secretion. Carnosine, assessed at different concentrations (from 200 nM to 200 mM), exerted an anti-cancer effect on breast (ZR-75-1), ovarian (SKOV-3), colon (HT29, LIM2045), and leukemic (U937) cancer cell proliferation. It also up-regulated the expression of pro-inflammatory molecules, modulated cytokine secretion, and altered U937′s differentiation and phenotype. The concentrations used in these studies are in line with those found endogenously in different human tissues (up to 20 mM) [86,87]. Furthermore, carnosine has been used at a high dosage (1500–2000 mg/day) in clinical trials to treat a variety of conditions, including type 2 diabetes, cardiovascular disease, stroke, and neurodegenerative diseases [88,89,90]. The cell specificity of carnosine’s inhibitory effects on distinct cell lines can be explained via metabolic differences between cell types, and more specifically whether they rely more heavily on glycolysis or mitochondrial adenosine triphosphate (ATP) synthesis for energy [91].

In a study carried out by Haabeth and collaborators, it was demonstrated that the enhanced gene expression of IL-1β can be decisive for the anti-tumoral action of carnosine [92]. IL-1β is a pro-inflammatory cytokine that has previously been proven to exhibit tumoricidal activity and to inhibit tumor growth. It is known that U937 cells, being a myeloid leukemia, have the capacity to secrete many cytokines and chemokines. The genes up-regulated in response to carnosine are largely inflammatory mediators; hence, they may contribute to its anti-cancer properties by making leukemic cells more detectable to the immune system. In addition, an increase in gene expression was observed for the chemoattractant *CCL2*, a small cytokine that belongs to the CC chemokine family known to exert both pro- and anti-tumoral effects [93]. There was also an increase in gene expression of the C-C chemokine receptor type 5 (*CCR5*), which binds different chemokines on the surface of white blood cells [94]. This protein may induce cancer, and in fact it has been used as a target in clinical studies [95]; however, some studies have shown anti-cancer effects as well. This paradoxical effect may be due to the type of cancer cells and the context in which the cancer cells originate [96]. Interestingly, the presence of carnosine with the up-regulation in *CCR5* might be involved in the anti-tumoral effects.

Another increase in terms of gene expression was observed with the complement *C3* and interferon regulatory factor 7 (*IRF-7*) in the presence of carnosine. While *C3* is generally known to promote cancer cell growth, several studies have demonstrated a contradictory role for *C3* and observed anti-cancer effects [97]. *IRF-7*, however, is known to decrease cancer growth and metastasis. In fact, silencing *IRF-7* in breast cancer cell lines increases growth, while restoring its expression reduces metastasis [98]. Similarly, in a mouse model of prostate cancer, metastasis was significantly reduced following the overexpression of *IRF-7* [99].

Through a cytokine assay, it was shown that carnosine increased the secretion levels of IL-10, GM-CSF, and TNFα, while decreasing IL-8. High levels of IL-10 lead to inhibition of tumor metastases, resulting in a slowdown of cell growth [100]. This is due to the increase in IFN-γ from CD8^+^ T cells [101], as also demonstrated in vivo in *IL-10* transgenic mice with stimulated CD8^+^ T cells, resulting in the limited growth of immunogenic tumor cells [102]. Despite IL-10 being known as an anti-inflammatory and immunosuppressive cytokine, it has also been shown to exert immunostimulatory activities. In fact, recombinant PEGylated IL-10 inhibited tumor cell growth in mice [103]. Therefore, it is reasonable to suggest that carnosine may exhibit anti-cancer activity in U937 cells by producing elevated levels of IL-10.

Another soluble factor, GM-CSF, a monomeric glycoprotein secreted by macrophages, T cells, mast cells, natural killer cells, endothelial cells, and fibroblasts, functions as a cytokine and is up-regulated by carnosine. GM-CSF controls cancer cell growth, leading to immunosuppression in the tumor microenvironment. Also, its increased secretion in the presence of carnosine contributes to its anti-proliferative potential. There are a number of clinical studies that have investigated the efficacy of direct injections of GM-CSF into the tumor. In addition, vaccines made using either GM-CSF fused with tumor-associated proteins or anti-cancer DNA with the incorporation of GM-CSF were successfully tested [104].

It has also been reported that carnosine can increase the expression and secretion of the pro-inflammatory cytokine TNFα, which is known to induce cancer cell death [105]. IL-8, best known for its immune chemo-attractive properties, also has a role in different human types of cancer. Particularly, it seems to be involved in metastasis and tumor progression by regulating cytokine secretion and angiogenesis [106]. In U937 cells treated with carnosine, secretion of IL-8 decreased, implying an inhibitory effect in cancer progression. The anti-cancer effects of carnosine on U937 cells stem from the simultaneous increased expression of IL-1β, increased secretion of IL-10, GM-CSF, and TNFα, and decreased secretion of IL-8.

**Figure 4:**
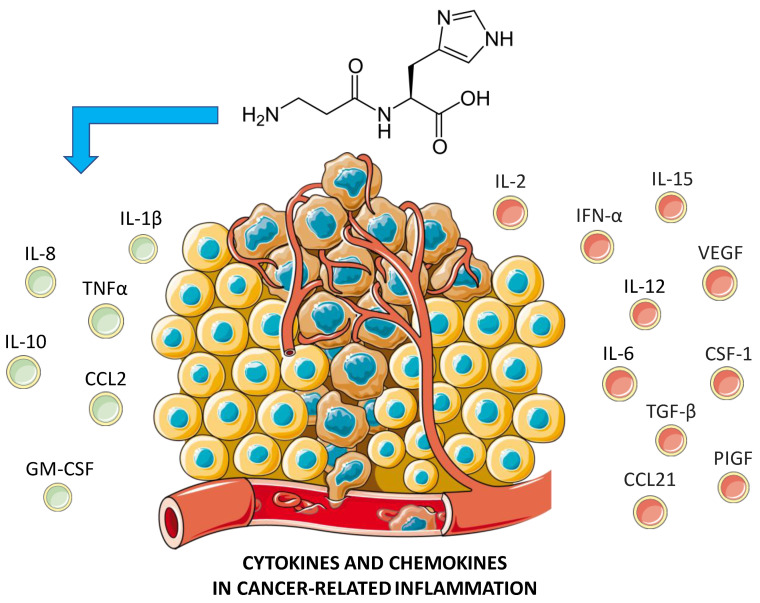
Cytokines and chemokines playing a role in cancer development and progression. The left panel refers to the cytokines and chemokines positively regulated by the dipeptide carnosine. Created with https://smart.servier.com (accessed on 24 September 2023).

In summary, carnosine has shown its ability to regulate the expression and/or secretion of different cytokines and chemokines directly related to cancer [107,108], while its possible modulatory activity on certain targets, such as IFN-α and IL-6, still need to be elucidated (Figure 4).

As previously mentioned, carnosine, among its various properties, exhibits antioxidant activity and is able to act against reactive oxygen species (ROS) [109]. In a study carried out by Reuter et al., carnosine’s antioxidant activity decreased oxidative stress and chronic inflammation, which are known hallmarks of cancer [110].

The effects of carnosine on gene expression are also worth further exploring. For example, some of the gene expression data showed that *IL-8*’s gene expression level is upregulated by carnosine [85], whereas other reports have shown a decrease in IL-8 secretion [111]. This discrepancy is possibly due to differences in the timing of measurements. The gene expression level was only measured 24 h after the administration of carnosine as compared to the measurement of cytokine secretion (5 days), which may account for these seemingly contradictory observations. Furthermore, it is also possible that the IL-8 protein was expressed but not secreted and was stored intracellularly instead, potentially requiring a second stimulus for secretion. Finally, in undifferentiated U937 cells carnosine treatment induced differentiation and phenotypic changes, with increased expression of CD11b, CD11c, CD86, and MHCII, thus enabling the promonocytic leukemia cells to be more visible to the immune system [85]. Another study investigated the anti-tumoral effects of carnosine, both in vitro and in vivo, and found that carnosine inhibited tumor-induced angiogenesis by reducing microvessel density in EJ(MGH-U1) cells (human bladder cancer) [112]. In a different study carried out by Lee et al., carnosine was able to induce apoptosis/cell cycle arrest via the suppression of the NF-κB/STAT1 pathway in HCT116 colorectal cancer cells [113].

### 4.2. Energy Metabolism/Bioenergetics

Recently, the importance of mitochondria as oxygen sensors, as well as producers of ATP, has become a fundamental point in cancer research, and studies have shown that mitochondrial metabolism is important for the rapid proliferation of several cancer cell types [114,115]. Therefore, mitochondria have been considered as a novel and effective therapeutic target for treatment in some types of cancer. The effects and mechanisms of carnosine on the viability and proliferation of the human gastric cancer cell line SGC-7901 were the focus of a study published by Shen et al. [46]. In this study, carnosine reduced the proliferative capacity of SGC-7901 without inducing cell apoptosis or necrosis. Measurements using a Seahorse XF96 extracellular flux analyzer showed that SGC-7901 cells cultured with pyruvate have active mitochondria and depend on mitochondrial oxidative phosphorylation more than the glycolysis pathway for the generation of ATP. Carnosine strongly decreased the absolute value of mitochondrial ATP-linked respiration and reduced the maximal oxygen consumption and spare respiratory capacity, which may reduce mitochondrial function correlated with proliferative potential. Carnosine also reduced the extracellular acidification rate and glycolysis of these cells. The fact that carnosine functions as a regulator of energy metabolism of SGC-7901, both for the anaerobic and aerobic pathways, suggests its therapeutic potential for gastric cancer therapy and lays the foundations for future preclinical and clinical studies.

Carnosine also exerts this effect in cervical cancer, which is the 3rd most common malignancy in women worldwide [116,117]. Specifically, the cell lines that were used were the human cervical gland carcinoma (HeLa) and the cervical squamous carcinoma (SiHa) cell lines. However, carnosine was only shown to be effective in HeLa cells, resulting in a significant reduction in glycolysis, mitochondrial bioenergetics, and proliferative capacity. In HeLa cells, carnosine decreased the activities of crucial metabolic enzymes, such as isocitrate dehydrogenase (IDH) and malate dehydrogenase (MDX) in the tricarboxylic acid cycle (TCA), instead of the electron transport chain (ETC) complexes I to IV. The TCA cycle, also known as the Krebs or citric acid cycle, is a major metabolic pathway that generates amino acid precursors (necessary for cell growth, repair, and proliferation), as well as NADH, which is an important reducing agent in oxidative phosphorylation. Interfering with any of the steps in the TCA cycle can result in altered mitochondrial bioenergetics [118]. Carnosine may also be able to disrupt mitochondrial protein homeostasis and respiration in HeLa cells by decreasing the activities of the ETC complexes I and II. This can be achieved through the down-regulation of caseinolytic protease proteolytic subunit (ClpP), one of the primary quality control proteases in the mitochondria. In a recent study, treatment with carnosine significantly reduced ClpP mRNA and protein levels in HeLa cells, resulting in a decreased proliferation through the inhibition of their mitochondrial metabolism. However, it is worth mentioning that the same effect was not observed in SiHa cells [119].

Table 1 reports the therapeutic effects of carnosine (outcomes), the metabolic pathways, and other relevant information related to carnosine in different types of cancer (including breast cancer), along with the related references.

## 5. Breast Cancer: Could Carnosine Exert a Therapeutic Effect?

With regard to breast cancer therapy, the approach currently used is multidisciplinary and includes surgery, radiotherapy, and chemotherapy, as well as the use of adjuvants, among others. The goal of an effective therapy should not only be to maximize therapeutic efficacy, but also be coupled with treatment strategies that minimize undesirable side effects [123]. Common systemic breast cancer drugs include bone-modifying agents, such as bisphosphonates, alkylating agents, anthracyclines, taxanes, aromatase inhibitors, selective estrogen receptor modulators, and immunotherapeutic agents, such as ERBB-targeted monoclonal antibodies [124]. It is crucial to understand that the selection of a specific medication varies when considering menopausal status and breast cancer stage.

There are also potential therapies that are still not approved, including proton therapy that offers advantages over photon therapy, in particular with regard to dose distribution. While the benefits and cost-effectiveness still need to be carefully evaluated, proton therapy has the potential to become a common radiotherapy modality for most types of solid cancer in the near future [125]. There has also been an increase in the development and usage of new oral-selective estrogen receptor degraders (SERDs) (e.g., AZD9496 and giredestrant (GD-9545)) and ER inhibitors (e.g., lasofoxifene and H3B6545). These molecules appear to be greatly bioavailable, have a higher therapeutic index, a better administration, and efficacy as compared to the current commonly used ER inhibitors [126]. Another class of molecules that has lately emerged as a promising prevention option for breast cancer is represented by plant-derived polyphenols (i.e., flavonoids) [127]. Flavonoids not only act through their antioxidant properties, but also interact with proteins. Furthermore, evidence supported by several epidemiological studies suggest that flavonoids can modulate tumor-associated macrophage function [128]. Despite this therapeutic promise, clinical applications in cancer trials are still limited due to their minimal dietary bioavailability.

Still in the context of new possible breast cancer treatments strategies, carnosine has been reported to exert a therapeutic effect on breast cancer cell proliferation [85]. A study conducted by Aydemir et al. investigated the potential anti-tumoral effects of carnosine in MCF7 cancer cells [129], a breast cancer cell line that was isolated in 1970 from a 69-year-old white woman [130]. The MCF-7 breast cancer cell line was cultured with carnosine (at dosages of 1 mM, 25 mM, 50 mM, or 100 mM) for 24 h, 48 h, and 72 h. The authors calculated the cell diameter for cells with and without carnosine at each time point. The 24 h- and 48 h-cultured MCF-7 cells treated with carnosine at all concentrations showed a reduced diameter and decreased cell viability when compared to the control, suggesting the ability of carnosine to inhibit the growth of this type of cancer in vitro. Further investigation into the effect of carnosine on these cells could include an additional experiment in which the treatment time is extended (e.g., up to 1 week); by doing so, it may be possible to understand whether an increase in the exposure time either results in increased anti-tumoral activity or it may result in a comparable therapeutic effect at lower dosages.

Interestingly, some of the current literature investigating carnosine’s therapeutic potential in breast cancer have featured a combination of carnosine with other delivery systems, such as carnosine-coated magnetic nanoparticles (CCMNPs), in the hopes of improving the chemotherapeutic efficacy of carnosine. When used on MCF-7 cells for 48 h, CCMNPs showed a 2.3-fold decrease in IC50 as compared to carnosine solution alone. Targeted delivery of carnosine in vivo was achieved by placing a magnet on the tumor site, resulting in a significant reduction in tumor size, while avoiding systemic toxicity. This treatment also resulted in a significant reduction in vascular endothelial growth factor (VEGF) levels as well as cyclin D1 levels. This targeted chemotherapeutic delivery system demonstrated a promising level of site-specificity capable of increasing efficacy, while minimizing undesirable systemic toxicity [120]. The increased anti-tumoral activity exerted by carnosine could also depend on its increased resistance to degradation by carnosinases when complexed to magnetic nanoparticles (CCMNPs).

In a different study, a PEGylated Liquisomes (P-Liquisomes) drug delivery system was employed with the aim of improving the anti-cancer activity of carnosine by prolonging its release and stability in vivo [121]. To avoid extensive degradation via carnosinases, carnosine was incorporated in a phospholipid complex in PEGylated liquid crystalline nanoparticles (P-LCNPs). The successful formation of the combined system was confirmed in vitro via the identification of spherical light-colored vesicles encapsulated in the liquid crystals. P-Liquisomes were characterized by an excellent complexation efficiency (97.5 ± 0.9%), also showing an impressive and sustained release of 75.4% (at 24 h), as compared to P-LCNPs or phytosomes. With regard to cytotoxicity, it was evaluated both in vitro and in vivo, showing different results. In the in vitro study, P-Liquisomes (IC50 = 25.9 mM) demonstrated a superior cytotoxic effect on MCF-7 cells after 24 h. When tested in mice induced with cancer, P-Liquisomes proved to be non-toxic and showed an enhanced activity by reducing tumor growth (−7.1%). It also decreased the levels of VEFG and cyclin D1, while increasing caspase-3 levels and the necrosis score [121].

In another recent study, Lei et al. developed a novel metabolic inhibitor called Zn–carnosine metallodrug network nanorods (Zn–Car MNs). Their anti-cancer activity was tested in vitro using a breast cancer cell line, namely 4T1, and in vivo on mice bearing 4T1 tumors or CT26 tumors [122]. It is known that breast cancer has a greater requirement for copper trafficking to the mitochondria compared to non-cancerous cells, especially due to the up-regulation of mitochondrial copper chaperones and cochaperones. Zn–Car MNs exhibited good Cu depletion and Cu-responsive drug release, causing potent inhibition of both the mitochondrial oxidative phosphorylation system (OXPHOS) and glycolysis. In particular, Zn–Car MNs were shown to reduce both cytochrome c oxidase activity and NAD^+^ levels, resulting in decreased ATP production in cancer cells. In turn, this resulted in a significant energy deprivation, leading to mitochondrial membrane depolarization, oxidative stress, and finally tumor cell apoptosis. Zn–Car MNs outperformed copper chelation by tetrathiomolybdate in in vivo breast cancer models, exerting the most effective antagonism of tumor growth. Of note, free carnosine was also able to restrict tumor growth compared to the vehicle group. Lastly, when investigated at the clinical level, a randomized phase III prospective placebo-controlled trial showed that Zn–carnosine was able to prevent dysphagia, the most common clinical manifestation in breast cancer patients undergoing adjuvant radiotherapy [131].

The previous results suggested that extracellular vesicles (EVs) may also be used as carriers for targeting tumors. EVs are lipid membrane nanoparticles released by cells in the surrounding microenvironment, where they transfer information (e.g., nucleic acids, proteins, and metabolites) from a donor to a specific target cell [132]. Interestingly, EVs from colon cancer cells treated with carnosine stimulated neurite outgrowth, thus suggesting the involvement of carnosine-induced EVs in the gut–brain axis [133]. These data support the potential of a carnosine–EV system as an advanced nanotherapy. In addition, recent studies have highlighted that tumor-derived EVs (TDEVs) were more likely internalized via the same type of tumor cells [134]. Therefore, opportunely engineered TDEVs, or hybrid vesicles, may be considered for the delivery of anti-cancer agents, including carnosine [135].

The prevention and/or counteraction of adverse effects from anti-tumoral therapies represents a mandatory step in the clinical setting, and therefore antidotes to these side effects could play a pivotal role in combination with supportive care [136]. Cisplatin is one of the most commonly used chemotherapy medications for the treatment of different tumors, but its use has been linked to nephrotoxicity [137,138]. Another drug, paclitaxel, is a taxoid chemotherapeutic agent, but it frequently causes painful peripheral neuropathy [139]. Both of these drugs have been extensively used for the treatment of breast cancer [138]. Interestingly, Zn–carnosine (also known as polaprezinc) has demonstrated the ability to reduce paclitaxel-induced peripheral neuropathy in rats without affecting ant-tumor activity [140]; furthermore, carnosine has been demonstrated to counteract oxidative stress on cisplatin-treated rats [141]. Further support for the possible use of carnosine as an antidote was shown in a randomized controlled clinical trial that showed that a modified bismuth quadruple therapy enriched with polaprezinc was more effective than a conventional triple therapy in eradicating *Helicobacter pylori*, while avoiding significant adverse events [142]. These studies support the possible use of carnosine as add-on therapy to mitigate the adverse effects of drugs (e.g., anti-tumoral drugs), while possibly enhancing the anti-cancer therapy thanks to its multimodal mechanism of action.

Nonetheless, one of the remaining challenges of using carnosine as a therapeutic agent could be represented by the presence of the CNDP1 and CNDP2 carnosinases, which are responsible for the minimal increase in circulating carnosine levels in humans after its administration, thus limiting the therapeutic potential of this dipeptide. It is possible that the highly promising results obtained in studies using mice and rats could represent an over-estimation of the therapeutic potential of carnosine, given that rodents do not express CNDP1 [4].

An additional factor to be taken into consideration are the possible side effects when using higher doses of carnosine. While the majority of pre-clinical studies have shown how this dipeptide is non-toxic [143] and extremely well tolerated by humans [144,145], with no known drug interactions, some studies have shown that carnosine (or its precursor, β-alanine) led to unwanted effects, such as anxiety-like effects in rats [146], and paresthesia [147] or increased perception of pain in humans [148].

## 6. Conclusions

Several studies have been carried out in the last few decades assessing the multimodal mechanism of action of carnosine, including its ability to modulate cell proliferation, cell cycle, apoptosis, and glycolytic energy metabolism, which are of main interest in the context of cancer.

In this review, we have examined the anti-tumoral activity that carnosine could exert in the context of different types of cancer, with a focus on breast cancer. In vitro studies showed that carnosine possesses the ability to decrease the size and viability of cancer cells. This dipeptide has also been proven to be able to reduce VEGF, cyclin D1, NAD^+^, and ATP levels, as well as cytochrome c oxidase activity. In a mouse model of breast cancer, carnosine was non-toxic for non-cancerous cells, while exhibiting chemopreventive activity observed via the reduction in tumor growth. When assessed at the clinical level in a randomized phase III prospective placebo-controlled trial, carnosine complexed to Zn was able to prevent dysphagia, the most common clinical manifestation in breast cancer patients undergoing adjuvant radiotherapy. These above-mentioned abilities support the anti-tumoral potential of carnosine, even though more preclinical and clinical studies are needed to support its therapeutic potential in treating breast cancer.

## Figures and Tables

**Table 1:** Anti-tumoral activity of carnosine in different cancer types.

Authors and Year	Cancer Cell Type/In Vivo Model	Effective Carnosine Dose	Time of Exposure	Outcomes	Ref.
Iovine et al., 2012	HCT116 (colon)	50–100 mM	24 h	Reduction in ROS levels and induction of cell cycle arrest in G1 phase; decreased ERK1/2 phosphorylation and increased p21waf1 protein; and reduction in ATP production via glycolysis.	[45]
Shen et al., 2014	SGC-7901 (gastric)	5–20 mM	24–48 h	Reduction in cell proliferation; decrease in mitochondrial ATP-linked respiration; reduction in maximal oxygen consumption and spare respiratory capacity; and reduction in the extracellular acidification rate and glycolysis.	[46]
Mikula-Pietrasik and Książek 2016	Patient-derived primary HPMCs (human peritoneal mesothelial cells)	20 mM	63 days	Delay of replicative senescence and inhibition of the development of two markers of senescence (i.e., SA-β-Gal and phosphorylated γ-H2AX); suppression of mitochondria-related oxidative stress; inhibition of adhesion, migration, invasion, and proliferation induced via down-regulated secretion of different molecules (i.e., fibronectin, IL-6, IL-8, GRO1, PAI1, and TGF-β1).	[47]
	A2780 (ovarian)	50 mM	24 h	Reduction in cell viability and increased percentage of apoptotic cells.	
	OVCAR-3 (ovarian)				
	SKOV-3 (ovarian)	100 mM			
Lee et al., 2018	HCT116 (colon)	100 mM or 200 mM	24 h	Reduction in cell proliferation by inducing the arrest in the G0/G1 phase; decreased mRNA levels of cell cycle-related genes; increased levels of the cyclin D1, BAX/Bcl-2, cleaved caspase-3, p21, and p53 proteins; and inhibited phosphorylation of STAT1 on Tyr701 and NF-κB p65 on Ser276 and Ser536.	[113]
Bao et al., 2018	HeLa (cervical gland)	20 mM	48 h	Inhibition of proliferation by inducing the arrest in the G1 phase; inhibition of both mitochondrial respiration and glycolysis, resulting in the reduction of ATP production; and decreased expression of ClpP protease.	[119]
Hwang et al., 2019	EJ cells (MGH-U1) (bladder)	≥20 mM	24 h	Inhibition of proliferation by blocking the G1 cell cycle phase; reduced levels of cyclin D1 and CDK4; dose-dependent increase in p21WAF protein expression; increased phosphorylation of ERK and dose-dependent reduction of p38; and inhibition of the migratory and invasive potential.	[112]
	Balb/C nude mice inoculated with EJ bladder cancer cells	5 mg/kg and 10 mg/kg	10 days	Reduction in the volume and weight of the tumor, without any loss of body weight as a side effect; and inhibition of angiogenesis.	
Farid et al., 2020	MCF-7 (breast)	1–100 mM used alone or conjugated with magnetic nanoparticles (CCMNPs)	24–48 h	CCMNPs displayed higher cytotoxic activity compared to the carnosine-free solution.	[120]
	Female BALB/C mice implanted with Ehrlich ascites carcinoma (EAC)	200 mg/kg/day (equivalent to 50 mM of carnosine)	21 days	Higher reductions in the volume and weight of the tumor in CCMNPs (both subjected or not to an external magnet placed on the tumor area) compared to the carnosine-free solution; and reduction in VEGF and cyclin D1 levels and increase in caspase 3 levels (increased apoptosis).	
Gaafar et al., 2021	MCF-7 (breast)	≥20 mM when incorporated into P-Liquisomes, phytosomes, and PEGylated liquid crystalline nanoparticles	24–48 h	Higher cytotoxic effect compared to carnosine alone, especially for the P-Liquisomes formulation.	[121]
	Female BALB/C mice implanted with Ehrlich ascites carcinoma (EAC)		3 weeks	P-Liquisomes induced: (i) the highest reduction in tumor volume, (ii) the major increase in the survival rate compared to the tumor-positive control; (iii) the most significant reduction in VEGF and cyclin D1 levels; and (iv) the highest level of caspase-3 activation.	
Prakash et al., 2021	U937 (myeloid leukemia)	≥100 mM	5–6 days	Inhibition of cell proliferation; up-regulation of pro-inflammatory molecule (IL-8, CCL2, CD86, IL-1β, CCR5, Ly96, IRF-7, C3, and TNF) expression; increased secretion of cytokines (IL-10, GM-CSF, and TNF-α); and stimulation of cell differentiation towards a macrophage phenotype.	[85]
	HT29 (colon)			Inhibition of cell proliferation.	
	LIM2045 (colon)				
	SKOV-3 (ovarian)				
	ZR-75-1 (breast)				
Lei et al., 2023	4T1 (breast)	≥20 µg/mL in the formulation Zn−carnosine metallodrug network nanoparticles (Zn–Car MNs)	24 h	Reduction of cell viability accompanied by the inhibition of both OXPHOS and glycolysis, resulting in a reduced ATP production; and increased ROS production and induction of apoptosis.	[122]
	Mice bearing 4T1 tumors	1 mg/mL in the formulation Zn−carnosine metallodrug network nanoparticles (Zn–Car MNs)	0, 2, and 4 days	Reduction in the volume and weight of the tumor, without body weight loss.	
